# Attitude towards Mental Illness among Secondary School Students in Asmara, Eritrea: A Cross-Sectional Study

**DOI:** 10.1155/2018/4578721

**Published:** 2018-11-01

**Authors:** Eyasu H. Tesfamariam, Medhane M. Tekie, Amos Y. Tesfa, Dawit H. Hadgu, Eyob A. Awalom, Eyob B. Ghebremedhin, Nebay A. Tquabo

**Affiliations:** ^1^Department of Public Health, Asmara College of Health Sciences, Asmara, Eritrea; ^2^Department of Nursing, Asmara College of Health Sciences, Asmara, Eritrea; ^3^Orotta School of Medicine and Dentistry, Asmara, Eritrea

## Abstract

Secondary School students (SSs) are important members of the community; hence their attitude towards mental illness can be highly influential. Mentally ill individuals are not only suffering from the illness but also suffering from the stigmatizing attitude generated by the community. The objectives of this study were to determine attitude of SSs towards mental illness and its associated factors. A cross-sectional study design employing stratified random sampling was applied to select a sample of 402 students. Data was obtained using a self-administered Belief towards Mental Illness (BMI) questionnaire. Independent sample t-tests and one-way ANOVA were used to determine possible differences in scores of attitude. From a total of 21 BMI scale items, positive attitudes were found in eight items and negative attitudes were found in the remaining thirteen. The mean score of the full BMI scale was 2.47 (95% CI: 2.41, 2.54). The mean (95% CI) scores of dangerousness, poor social relations and incurability, and shame subscales were 2.68 (2.60, 2.76), 2.55 (2.48, 2.62), and 1.22 (1.09, 1.34), respectively. A significant negative correlation was found between attitude scores and the average mark of students (r = -0.257,* p*<0.0001). Moreover, significant differences in attitude scores were observed between students with a relative of mental illness and those without such a relative (*p*=0.004). There was an increasing trend of positive attitudes with increased educational level among 9th, 10th, and 11th graders (*p*-trend<0.0001) and with an increase in the educational level of the students father (*p-*trend=0.028). However, no significant difference in attitude score was found across categories of sex, religion, living condition of father, presence of a mentally ill neighbor, educational level of mother, or ethnicity. In conclusion, considerable numbers of SSs have negative attitudes towards mental illness. Implementation of programs that enhance positive attitudes towards mentally ill individuals is recommended.

## 1. Introduction

Mental illness (MI) refers to mental and emotional impairments; it also comprises mental retardation, organic brain disease, and learning disabilities [1]. It can occur to any person without regard to personal characteristics. Globally, mental illnesses and their complications are common and leading burdens of health, with more than 600 million people suffering with depression and anxiety, which are the most common types of mental illness [2]. According to a WHO report, in any given year 8.25% to 29.1% of individuals are mentally ill and life time prevalence ranges from 12.2% to 48.6% [3]. The WHO predicts that mental illness will increase among teenagers by 50% until 2020 [1].

Studies have revealed that half of chronic mental illnesses occur by age of 14, and three-quarters by age of 24 [4]. One-fifth under 18 years old are diagnosed with some form of developmental, emotional, or behavioral problems [5]. Moreover, the proportion of mental illness in younger age groups ranges from 3% to 12% [1]. Hence, given the prevalence of mental illness and the increasing trends associated with the problem, studies that mainly focus on young people are important. Various studies that focus on young people can be conducted; however, studies regarding how mentally ill individuals are accepted without being stigmatized by youngsters are especially important.

A good picture of young people's attitudes towards mental illness can be obtained from Secondary School students (SSs). The attitudes of SSs are highly influential to the community. For instance, it has been found that providing health education concerning mental illness to SSs improved their understanding about the illness, as well as to their community [6]. This in turn was found to contribute towards successful treatment, social integration, help seeking behavior, and adherence to drug treatments [7].

The Ministry of Health of Eritrea and WHO in 2006 reported that there were about 33,000 “patient contacts” in the outpatient department of the only psychiatric hospital in Eritrea per year [8]. The diagnoses, in accordance with International Disease Classification (ICD 10), of new patients in 2002 were schizophrenia (11%), affective disorder (17%), neurotic and stress related disorder (22%), unclassified behavioral mental disorder (15%), mental retardation (3%), epilepsy (14%), dementia (4%), headache (10%), and being not diagnosed (4%). The dominant admission diagnoses, based on ICD 10, for 2004 were schizophrenia (32%), disorders of adult personality and behavior (25%), and affective disorders (21%) [8]. In a very small country with such mental health profile, it is important to obtain information concerning stigmatizing attitudes of youngsters emanated from ancient misconceptions.

Secondary School students as influential members of society need to be built up in all aspects of life, one of which is acceptance attitude towards mentally ill persons. This study, the first of its kind in the country, was conducted to assess the attitude of SSs towards mental illness and its possible associated factors. Reducing stigma can contribute towards protecting the human rights of individuals affected by mental illnesses [8].

## 2. Methods

### 2.1. Study Design and Setting

A cross-sectional study was undertaken among SSs of Asmara the capital of Eritrea, from 1^st^ of March to 30^th^ of April 2017. The approach was quantitative. The projected population size for 2017 is 804,000 with an area of 44.97 km^2^ at an elevation of 2,325 meters above sea level. The Secondary Schools in Asmara are Adulis, Asmara Comprehensive, Barka, Dembe Sembel, Halay, Harnet, Isaac Teweldemedhin, Keih Bahri, Lmaet, Semaetat, and Sembel.

### 2.2. Sampling Techniques and Sample Size

Samples were obtained using stratified random sampling. The sampling frame consisted of the list of all 19,200 students in 11 Secondary Schools, which were considered as strata. The minimum sample size assuming a 95% level of significance, 5% error, and 50% expected proportion was 385. However, a population correction factor was introduced reducing the sample size to 377. After 6% adjustment for nonresponders and missing students, the final sample size was 402.

### 2.3. Sample Allocation

Samples were taken proportional to the population size of the respective school (Table 1). Students were enrolled systematically from each school.

### 2.4. Data Collection Tool

A self-report questionnaire consisting of questions regarding demographic characteristics and attitude towards mental illness of the students was developed. The demographic characteristics consisted of age, sex, grade of the students, address, ethnicity, father's and mother's educational level, living condition of father, religion, existence of mental illness in the student's family, and existence of mental illness among the student's relatives or neighbors. The questionnaire used to assess the attitude towards mental illness was a standard questionnaire with accepted reliability and validity, the so-called Belief towards Mental Illness (BMI) [9]. BMI uses a 6-point Likert-type scale and was scored as 0 = completely disagree, 1 = largely disagree, 2 = partly disagree, 3 = partly agree, 4 = largely agree, and 5 = completely agree. The full-questionnaire contains 21 items with three subscales. The first subscale, “dangerousness”, measures how dangerous mental illness and mentally ill individuals are using eight items. The second subscale was “incurability and poor social and interpersonal skills” that measures how people think mental illness affects interpersonal relationship and its incurability using eleven items. The third subscale, “shame”, measures how individuals feel ashamed about mental illness using two items. The scale is interpreted according to both total scores and subscale scores. High scores represent negative beliefs. Factor analysis from a previous study in Turkey, which utilized the same questionnaire, clearly determined its three subscales with Cronbach-*α* ranging from 0.69 to 0.80 and satisfactory overall Cronbach-*α* of 0.82 [10]. Besides, the internal consistency in our study has been computed and found to be satisfactory (Cronbach-*α* = 0.736). Subscale wise, the Chronbach-*α* of shame, dangerousness, and incurability and poor social and interpersonal skills were 0.441, 0.643, and 0.685, respectively.

### 2.5. BMI Tool Translation and Data Collection Procedure

The original questionnaire was in English but it was translated into a local language, Tigrigna, to be easily understood by the students. The consistency of the translated version was confirmed through back translation. It was pretested to assess its clarity and intelligibility in 30 randomly selected students (not part of the sampled students in the actual study) at three schools. The appropriate time needed to fill the questionnaire by the students was also determined in the pilot study. Hence, the randomly selected Secondary School students were given 20 minutes to fill the questionnaires after their class break. Instructions, with regard to honesty as well as not skipping any question while responding, were given to the students.

### 2.6. Data Analysis

Data was double entered in Census and Survey Processing system (CSPro, Version 7) to minimize entry errors and then exported to Statistical Package for Social Sciences (SPSS Inc., Chicago, Illinois, USA, version 22) for analysis. Descriptive analysis was done using frequency (percentage) for categorical variables and mean (standard deviation) for quantitative variables. Normality of the attitude scores was checked using Kolmogrov-Smirnov and Shapiro-Wilk's tests. After confirming normality, inferential analysis was conducted using Pearson's correlation coefficients, t-tests, and one-way analysis of variance (ANOVA) in order to make comparisons among different categories of the predictor variables.* P-*values < 0.05 were considered to be significant in all the analyses.

## 3. Results

### 3.1. Demographic Characteristics of Sample Study

The sample was comprised of two hundred and twenty (54.7%) females and 182 (45.3%) males (Table 2). The age of the respondents ranged from 13 to 20 years with a mean of 15.81 (SD=1.12 years) and the mean and SD of the students' semester marks were 71.20 and 11.26, respectively (range: 32.00 to 97.50). The distribution of students by grade was similar between grades 9 (31.84%), 10 (35.32%), and grade 11 (32.84%). The majority of the respondents were Christian (90.30%) and ethnic Tigrigna (94.28%). The percentage of students in which their fathers' educational level was elementary and below were minimum (12.94%), while the percentage of students whose fathers were tertiary level were maximum (48.01%). However, the highest percentage (31.59%) of mothers' educational level was Secondary School and the lowest percentage (19.15%) Junior School. The majority (97.01%) of students had no family history of mental illness, and 28.8% of them had a neighbor with a history of mental illness.

### 3.2. Attitude of the Students towards Mental Illness

#### 3.2.1. Attitude by Item

A higher percentage of positive than negative attitudes were observed in eight out of twenty-one of the evaluated BMI scale items (Table 3). On the other hand, the students had a higher percentage of negative attitudes in the remaining thirteen BMI scale items.

#### 3.2.2. Attitudes by Subscale

The mean for each subscale was determined in a way that helps to assess at which level of agreement the students were with the negatively coined BMI items. The computed mean corresponds with the Likert scale scores assigned originally to each item. The mean score of the dangerousness subscale among the SSs was 2.68 (95% CI: 2.60, 2.76) (Table 4). The mean for poor social relations and incurability was found to be 2.55 (95% CI: 2.48, 2.62). The third subscale, shame, had a mean of 1.22 (95% CI: 1.09, 1.34). The mean (95% CI) of the full BMI scale was 2.47 (2.41, 2.54).

#### 3.2.3. Associates of Attitude

The possible association of the attitude score with the average mark of the students was checked using Pearson's correlation coefficient. On the other hand, the possible differences in the attitude scores among various independent variables having two categories were determined using independent sample t-tests. Finally, differences in attitude scores for those independent variables having more than two categories were investigated using one-way ANOVA.

There was a significant negative correlation between the attitude scores and average mark of the students (r = -0.257,* p*<0.0001). Independent sample t-test revealed that sex (*p*=0.890), religion (*p*=0.310), living condition of the students father (*p*=0.650), presence of a neighbor with mental illness (*p*=0.420), and ethnicity (*p*=0.210) did not affect attitude (Table 5). Students who have relative with mental illness had significantly higher positive attitude than those who did not have such a relative (*p*=0.004).

There was significantly higher positive attitude towards mental illness among 11^th^ graders than 10^th^ and 10^th^ graders than 9^th^  (*p*<0.0001) (Table 6). An increasing trend of positive attitudes with an increase in educational level among 9th, 10th, and 11th graders (*p*=0.0001) was also observed. Similarly, a significant difference in attitude according to the educational level of the student's father (Illiterate, Elementary, Junior, Secondary School, and Tertiary level) (*p*=0.020) was found. In addition, the increase in educational level of the student's father was associated with an increase in students' positive attitude (*p*-trend=0.028). On the other hand, no significant difference in attitude scores was observed among the students with an increase in the educational levels of their mothers (*p*= 0.110).

## 4. Discussion

A community's perception of mental health varies across cultures as a result of which different myths and beliefs emerge [11]. Most mental health related surveys have been largely conducted in western countries, with only a few studies in developing countries. For this reason, the factors that have led to the variety in attitude differ from one country to another and from generation to generation through several ways such as observation, operant conditioning, and cognitive learning. This research paper, using the BMI scale, describes the attitude towards mental illness and associated factors among Secondary School students in Asmara.

The BMI scale, having three subscales, was first designed by Hirai and Clum to assess negative views of mental illness. The three subscales of the instrument are dangerousness, poor social and interpersonal skills, and incurability. Each of the subscales has their own scores depending on the number of items, which upon totaling gives the overall BMI scale's score. BMI scale does not have a cut-off point for separating the various possible categories of attitude. However, as the scores increase the negative attitude towards mental illness also increases. The full scale BMI scores have shown that a typical Secondary School student has an ambivalent attitude towards mental illness. The possible reason might be the cumulative effect of stigmatizing negative attitudes learned from elders or reinforced by sociodemographic features. Another study using a different scale conducted among Nigerian Secondary School students showed that just over half (55%) of the students had a positive attitude towards mental illness [12]. Negative attitudes were not only present in Secondary Schools but were also found among university students in Pakistan [13]. Suggested reasons for negative attitudes have been the style of upbringing [13] and insufficient information of the illness [14].

In addition to the acceptable reliability and validity of the BMI scale by the designers in 2000, sound psychometric properties were also found with regard to substantive, content, structural, and generalizable aspects of validity using Mesick's framework in 2013 [15]. In this study, subscale analysis of BMI for the dangerousness as well as poor social and incurability subscales revealed that a typical student had attitudes approaching the negative. This could be due to a lack of adequate knowledge about different types of mental illnesses [16]. Besides, continuous observation of chronically sick individuals in the streets harming others could be another contributor to dangerousness and incurable attitudes. This demands the need for attitude enhancing programs. More positive and favorable attitudes were seen in the shame subscale. However, the fact that only two questions in the BMI scale constitute the shame subscale need to reminds us that the results must be considered with caution.

It was realized, from the response to the statement* “A mentally ill person is more likely to harm others than a normal person”,* that students had negative attitude because 76% of them agreed in their response. A study conducted among college students in India also showed a relatively higher percentage (90%) of negative attitudes for the same item [16]. One reason for this negative attitude in Eritrean students could be the failure to provide educational programs concerning mental illnesses. Another considerable negative attitude observed was that 82% of the students agreed that mentally ill individuals have difficulty to follow social rules, such as being punctual or keeping promises. However, this might have been an assumption based on a lack of practical experience that might have developed from looking at chronically ill individuals commonly seen on the streets.

On the other hand, more students had found positive attitudes on certain items, some of which were embarrassment if family member gets ill (83%), embarrassment upon dating a mentally ill person (78%), and trust in the work of a mentally ill person (76%). The positive attitude on these three items could be due to the increased contact with mentally ill people in and around the places that they live or work. Such contact tends to diminish negative attitudes towards the illness [17]. Besides, more students were also found to have positive attitude on effectiveness of medical treatment for mental illness (65%). A similar result was obtained from a study conducted in Nigerian Secondary School students in which 79% had positive attitude on effectiveness of medical treatment from mental illness [12]. Current medical advances and their curative abilities could be the reason for the positive attitude regarding effectiveness of medical treatment among the students. This is in contrast to the 94% of college students in India who considered that mental illness was incurable [16].

In our study, grade eleven students had a more positive attitude towards mental illness than grades 9 or 10 students. In line with this, a report written in Nigeria has revealed that attitude towards mental illness has significant association with educational level [18]. Similarly, individuals with low educational level had lower acceptance and more stigma towards the illness [19].

Having a mentally ill relative was found to be associated with a more positive attitude towards mental illness. This finding is in congruence with a study done by Corrigan which demonstrated that existence of positive interaction with individuals affected by mental illness can change negative attitudes and perceptions towards the disease [20]. Nevertheless, presence of a mentally ill individual in the neighborhood was not significantly associated with attitude towards mental illness.

Even in this modern era, mentally ill individuals are not free from discrimination and stigmatization by the public. Negative attitudes and stigmatization are widespread towards mentally ill individuals. The negative attitudes of students might lead to withdrawal of the mentally ill individuals from social interactions and aggravate the illness. Hence, intervention that increases knowledge, which in turn reduces stigmatizing attitude, is required.

## 5. Conclusions

A considerable number of students in Secondary Schools of Asmara had negative attitudes towards mental illness. Yet, progress in educational level can inculcate a positive attitude and perception towards mental illness. Moreover, having a relative with mental illness contributes towards a positive attitude concerning mental illness.

Mental health professionals need to implement attitude enhancing programs and provide relevant information to Secondary School students. The media, through launching specific programs relevant to mental illnesses, should play their role in reducing the stigmatizing attitudes of the community at large especially of students. Other complementary qualitative researches are also recommended to discover further aspects of attitudes towards mental illness.

The results of this study will help the Ministry of Health and Ministry of Education to target interventions on attitudes towards mental illness among Secondary School students. In addition, these results contribute to the literature on attitude towards mental illness using a reliable and valid BMI scale by portraying the possible differences and similarities across cultures and countries.

## Figures and Tables

**Table 1:** Sample allocation of students by school (n=402).

**School Name**	**School Size**	**Proportion Allocated**	**Total Sample**
Adulis	1289	0.0671	27
Asmara Comprehensive	2189	0.1140	46
Barka	2052	0.1069	43
Dembe Sembel	642	0.0334	13
Halay	2539	0.1322	53
Harnet	1755	0.0914	37
Isaac Teweldemedhin	1822	0.0949	38
KeihBahri	2400	0.1250	50
Lmaet	1267	0.0660	27
Semaetat	1831	0.0954	38
Sembel	1414	0.0736	30

**Total**	**19200**	**1**	**402**

**Table 2:** Distribution of the study sample by socio-demographic characteristics (n=402).

**Characteristics**			**Number (n)**	**Percent (**%**)**
**Grade**				
	Grade 9		128	31.84
	Grade 10		142	35.32
	Grade 11		132	32.84
**Sex**				
	Male		182	45.27
	Female		220	54.73
**Religion**				
	Christian		363	90.52
	Islam		35	8.73
	Other		3	0.75
**Ethnicity**				
	Tigrigna		379	94.28
	Others‡		23	5.72
**Father's Education level**				
	Elementary and Below		52	12.94
	Junior		72	17.91
	Secondary School		85	21.14
	Tertiary Level		193	48.01
**Mother's Educational level**				
	Elementary and Below		102	25.37
	Junior		77	19.15
	Secondary School		127	31.59
	Tertiary Level		96	23.88
**Family History of MI**				
	No		390	97.01
	Yes		12	2.99
**Neighbor History of MI**				
	No		286	71.14
	Yes		116	28.86

	Minimum,	Maximum	Mean	SD
**Age**	13	20	15.81	1.13
**Average Score**	32	97	71.2	11.26

‡Others include Tigre (n=12), Saho (n=6), Blien (n=3), Afar (n=1), and Rashaida (n= 1).

**Table 3:** Percentage of positive and negative attitude for the BMI scale items.

**Item **	**Positive Attitude n (**%**)**	**Negative Attitude n (**%**)**
**1.** It may be a good idea to stay away from people who have psychological disorder because their behavior is dangerous	226 (57)	176 (43)
**2.** A mentally ill person is more likely to harm others than a normal person	98 (24)	304 (76)
**3**. Mental disorders would require a much longer period of time to be cured than would other general diseases	70 (17)	332 (83)
**4**. I would not trust the work of a mentally ill person assigned to my work team	306 (76)	96 (24)
**5**. The term “psychological disorder” makes me feel embarrassed	239 (59)	163 (41)
**6.** A person with psychological disorder should have a job with only minor responsibilities	140 (35)	262 (65)
**7.** Mentally ill people are more likely to be criminals	168 (42)	234 (58)
**8.** Psychological disorder is recurrent	112 (28)	290 (72)
**9.** I am afraid of what my boss, friends, and others would think if I were diagnosed as having a psychological disorder	204 (51)	198 (49)
**10.** Individuals diagnosed as mentally ill suffer from its symptoms throughout their life	161 (40)	241 (60)
**11.** People who have once received psychological treatment are likely to need further treatment in the future	76 (19)	326 (81)
**12.** It might be difficult for mentally ill people to follow social rules such as being punctual or keeping promises	74 (18)	328 (82)
**13.** I would be embarrassed if people knew that I dated a person who once received psychological treatment	315 (78)	87 (22)
**14.** I am afraid of people who are suffering from psychological disorder because they may harm me	182 (45)	220 (55)
**15.** A person with psychological disorder is less likely to function well as a parent	157 (39)	245 (61)
**16.** I would be embarrassed if a person in my family became mentally ill	332 (83)	70 (17)
**17.** I believe that psychological disorder can never be completely cured	266 (66)	135 (34)
**18.** Mentally ill people are unlikely to be able to live by themselves because they are unable to assume responsibilities	144 (36)	257 (64)
**19.** Most people would not knowingly be friends with a mentally ill person	134 (33)	268 (67)
**20.** The behavior of people who have psychological disorders is unpredictable	96 (24)	306 (76)
**21.** Psychological disorder is unlikely to be cured regardless of treatment	263 (65)	139 (35)

**Table 4:** Mean (SD) and 95% CI for the three subscale and overall score.

**Subscale **	**M (SD)**	**95**%** CI**
Dangerousness	2.68 (0.80)	2.60, 2.76
Poor social relation and incurability	2.55 (0.73)	2.48, 2.62
Shame	1.22 (1.30)	1.09, 1.34

**Full BMI scale **	**2.47 (0.65)**	**2.41, 2.54**

**Table 5:** Difference in attitude scores by sociodemographic characteristics.

**Characteristics**	**M(SD)**	**Diff.** *∗ * **(95 CI)**	***p*-value**
**Sex**			
Male	52.00(12.93)	0.18 (-2.50,2.89)	0.890
Female	51.82(14.19)		
**Religion**			
Christian	52.17(13.57)	2.45 (-2.26,7.16)	0.310
Islam	49.72(13.03)		
**Father Alive**			
Yes	51.99(13.84)	1.23 (-3.95,6.39)	0.650
No	50.76(10.75)		
**A Relative with Mental Illness**			
Yes	49.66(14.06)	3.06 (0.06,6.07)	0.040
No	52.72(13.39)		
**A Neighbor with mental illness**			
Yes	52.77(14.27)	1.22 (-4.18,1.73)	0.420
No	51.55(13.37)		
**Ethnicity**			
Tigrigna	52.10(13.47)	3.66 (-2.07,9.93)	0.210
Others	48.44(15.92)		

*∗*Diff.= difference in mean.

**Table 6:** Difference in attitude towards mental illness by educational level of student and educational level of father and that of mother.

**Characteristics**	**M(SD)**	**F- value**	***P*-value**	***P*-value trend**
**Educational Level of Student **				
Grade 9	55.02(14.46)	9.09	<0.0001	<0.0001
Grade 10	52.63(12.71)			
Grade 11	48.11(12.91)			
**Educational Level of Father **				
Elementary or below	53.66(15.26)	4.70	0.001	0.005
Junior	55.87(11.98)			
Secondary School	50.72(13.59)			
Tertiary Level	50.45(13.49)			
**Educational Level of Mother **				
Elementary or below	54.53(14.42)	1.96	0.120	0.200
Junior	49.85(12.84)			
Secondary School	51.33(13.32)			
Tertiary Level	51.44(13.53)			

## Data Availability

The data used to support the findings of this study are available from the corresponding author upon request.

## Abbreviations

SSs: Secondary School students
ACHS: Asmara College of Health Sciences
ANOVA: Analysis of variance
BMI: Belief towards mental illness
SPSS: Statistical Package for Social Sciences
M: Mean
CI: Confidence interval
MI: Mental illness
WHO: World Health Organization
ICD (10): International Disease Classification
SD: Standard deviation
Diff.: Difference in mean.
